# On the way to sustainability: the role of green marketing strategies and best practices of TQM on green performance among nurse managers

**DOI:** 10.1186/s12912-025-03714-5

**Published:** 2025-09-10

**Authors:** Manal Saleh Moustafa Saleh, Riham Hashem Fathi, Sahar Mohammed Mohammed Aly, Mohamed Zoromba, Sahar Abdel-Latif Abdel-Sattar, Hamad Ghaleb Dailah, Abdulqadir J. Nashwan, Hanan Elsaid Elsabahy

**Affiliations:** 1 Department of Nursing Sciences, College of Applied Medical Science, Shaqra University, Shaqra, Saudi Arabia; 2 Nursing Administration, Faculty of Nursing, Zagazig University, Zagazig, Egypt; 3 Faculty of Medicine, Zagazig University, Zagazig, Egypt; 4 Nursing Leadership and Management, Nursing College, Sulaiman Al-Rajhi University, Al Bukairiyah, Saudi Arabia; 5 Nursing Administration, Faculty of Nursing, Port Said University, Port Said, Egypt; 6 College of Nursing, Prince Sattam Bin Abdulaziz University, Al Kharj, Saudi Arabia; 7 Nursing Department, College of Nursing and Health Sciences, Jazan University, Jazan, Saudi Arabia; 8 Nursing & Midwifery Research Department, Hamad Medical Corporation, Doha, Qatar; 9 Nursing Administration, Faculty of Nursing, Mansoura University, Mansoura, Egypt

**Keywords:** Nurse managers, Sustainability, Green marketing strategy, TQM, Green performance

## Abstract

**Background:**

Any hospital’s ability to successfully provide healthcare services depends heavily on its nurse managers. They comprise a larger portion of healthcare institutions. There are three things to blame for the problems hospitals face today: poor performance, a lack of dedication among nurses, and poor TQM. The study aligns with Saudi Vision 2030, which emphasizes sustainability as a crucial component of the country’s development. Nurse managers can improve green performance by incorporating Total Quality Management (TQM) best practices and green marketing strategies.

**Aim:**

This study is designed to evaluate the impact of green marketing strategies and best practices of TQM on green performance among nurse managers.

**Method:**

A quasi-experimental design with pre- and post-tests was conducted with 32 nurse managers who completed the intervention. Data were collected using the Green Marketing Knowledge Questionnaire, Green Marketing Practice Scale, TQM Questionnaire, and Nurse Manager Performance Assessment Scale.

**Results:**

The green marketing strategy educational intervention led to significant improvements across all measured domains. Nurse managers’ knowledge of green marketing increased from a median score of 6 to 15. Similarly, the total green marketing practice score improved from 251 to 352, while TQM practices and green performance also showed notable enhancements. Post-intervention, TQM practice median scores increased from 125 to 188.5, and green performance scores improved from 71to 139. These improvements persisted three months after the intervention, indicating the sustained impact of the program.

**Conclusion:**

The findings demonstrate that training in green marketing strategies equips nurse managers with the knowledge and skills to enhance TQM practices and green performance, ultimately contributing to organizational sustainability. The study underscores the importance of integrating green marketing strategies into hospital management practices to address environmental and performance challenges.

**Implications:**

This study contributes to the growing body of evidence supporting the integration of sustainability-focused interventions in healthcare. It highlights the role of nurse managers in driving green performance and promoting a culture of sustainability within healthcare organizations.

**Clinical trial number:**

Not applicable.

## Introduction

Nurse managers are essential for sustainable practices to be successfully implemented in healthcare settings. Nurses control day-to-day healthcare operations, handle medical supplies, and have direct patient interaction. Reducing the environmental impact of healthcare facilities requires their participation in eco-friendly activities, such as appropriate waste disposal, energy conservation, and resource efficiency. Nursing professionals can significantly contribute to the advancement of sustainability goals while upholding the highest standards of patient care by implementing green marketing techniques and TQM best practices [1]. An essential part of putting green marketing concepts into practice is nurse managers. Using environmentally friendly medical products and energy-saving techniques directly contributes to the institution’s green performance [2].

In recent years, the integration of green marketing strategies with total quality management (TQM) practices has emerged as a pivotal approach for enhancing environmental sustainability within healthcare settings. Nurse managers play a crucial role in this integration, as they are responsible for implementing policies that comply with environmental regulations and promote a culture of sustainability among their teams [3]. By leveraging green marketing strategies, nurse managers can effectively communicate the benefits of eco-friendly practices to staff and patients alike, fostering a commitment to environmental stewardship. Simultaneously, TQM principles, such as continuous improvement and customer satisfaction can be tailored to emphasize sustainable practices, thereby enhancing operational efficiency and reducing waste. Together, these strategies not only improve green performance but also contribute to a more sustainable healthcare environment, aligning with the broader goals of public health and community well-being [4, 5].

In reaction to the growing consumer demand for eco-friendly products, “green marketing strategy” has become one of today’s most successful corporate approaches [6]. Five factors contribute to the increased usage of green marketing to address these strategies and intervention programs. First, businesses view environmentally responsible marketing as a tool for accomplishing objectives. Second, organizations think it is morally required of them to take greater social responsibility. Third, the government established rules mandating that homeowners take greater responsibility for the environment. Fourth, managers are forced to alter their marketing strategy by rival hospitals that have adopted green marketing first. Ultimately, hospitals are forced to adjust their strategy and practices due to the financial implications of disposing of waste or using less materials [7].

The concept of green marketing holds significant value, even though it does not directly refer to “green pricing.” It comprises using marketing strategies to promote goods and services that are friendly to the environment. Green marketing recognizes that certain consumers are willing to pay extra for products that reduce their environmental impact. Using recycled materials, cutting energy use, and limiting environmental effects are just a few examples of how businesses may use green marketing to highlight the environmentally friendly features of their goods and services. Green marketing, on the other hand, can encourage the use of TQM techniques by coordinating quality targets with sustainability goals [8].

According to the definition of TQM, organizations continuously strive to either meet or surpass their clients’ demands and expectations, whether they are internal or external. TQM is a system that an organization’s management implements to attain patient/customer satisfaction [9]. In this age of globalization, TQM has become increasingly important as a strategy to enhance organizational performance. Several studies have demonstrated how TQM may improve system quality and boost employee and organizational performance responsibilities [10]. TQM stands for complete quality control, quality management, and continual improvement. TQM is regarded as an avant-garde method of managing businesses. Quality orientation is integrated into all aspects of TQM in the medical field [11].

In addition, the green performance of nurses is crucial to ensuring that patients receive the high-quality care they need in the clinic. A task’s performance is determined by comparing it to predetermined benchmarks for speed, accuracy, completeness, and cost. Employee green performance is measured by their output in terms of quantity, quality, punctuality, and attendance at work, as well as by how effectively and efficiently they execute their work. The most important objective of every nurse management is to provide high-quality patient care on the unit, and the head nurse has been defined as essential to achieving this goal. In the patient care division, the primary duty of head nurses is to ensure that all patients’ requirements are satisfied; hence, all unit activities should be directed towards this goal [12, 13]. Three criteria are used to evaluate head nurses’ green performance. First, general traits including looks, work habits, and timeliness are utilized to assess a head nurse’s job green performance. Second, soft skills including teamwork among employees, communication with patients, creativity, documentation, and technical upkeep. Third, nursing care, which covers patient safety, preventative measures, and activities in the nursing care plan [14].

Enhancing customer satisfaction and environmental quality are the two objectives of the training program that supports the green marketing strategy. Therefore, incorporating green marketing into a marketing plan may be essential to hospitals gaining a long-term competitive edge. The hospital marketing team currently employs green marketing as a primary approach for sustainable competitive advantage SCA [15]. Thus, the main goal of this study was to determine whether nurse managers’ knowledge of green marketing can be increased by green marketing strategy intervention to fill in these gaps.

Teaching nurse managers about green marketing techniques could have a lot of advantages. Training in green marketing strategies has previously improved an organization’s performance and corporate image [16]. The purpose of the current study was to close these gaps by examining the relationship between green marketing strategies TQM best practices and green performance among nurse managers. The researchers were unable to find any published studies on this topic, so this study’s secondary goal was to do just that. The outcome of this study has the potential to support the kingdom’s commitment to a more sustainable and greener future by promoting eco-friendly healthcare practices, encouraging a culture of sustainability, and stimulating innovation in nursing leadership.

In light of the above, this study aims to evaluate the impact of green marketing strategies and best practices of Total Quality Management (TQM) on green performance among nurse managers within the framework of sustainability. Specifically, the study seeks to answer the following research questions:

1. Will nurse managers demonstrate higher levels of green marketing strategy and its related dimensions after participating in the green marketing strategy intervention compared to before the intervention?
2. Will nurse managers engage in a higher level of Total Quality Management (TQM) after the green marketing strategy intervention compared to their engagement before the intervention?
3. Will nurse managers show improved levels of green performance after participating in the green marketing strategy intervention compared to the pre-intervention levels?

The remainder of this paper is structured as follows: Sect. “Theoretical framework” presents the theoretical framework and research hypotheses. Section “Subjects and methods” describes the methodology, including design, sample, and intervention. Section “Results” outlines the results of the hypothesis testing. Section “Discussion” discusses the findings in light of existing literature. Finally, Sect. “Conclusion and recommendations” provides the study’s conclusions, implications, and recommendations for future research.

## Theoretical framework

Green marketing, also known as environmental marketing or sustainable marketing, has become an important strategy in today’s ecologically conscious society. The results of interventions using green marketing strategies have been examined in earlier research. The impact of green marketing on customer satisfaction and retention in the Indian auto sector was examined by [17]. The study discovered a link between green marketing and customer satisfaction, which in turn boosts customer loyalty [18, 19]. With a focus on developing and promoting ecologically friendly products that adhere to several standards, green marketing has emerged as a significant idea in the modern market [20].

Research indicates that structured educational interventions improve knowledge, which influences behavior and performance. Nurse managers, who are key decision-makers, can only successfully implement green marketing if they thoroughly understand its principles and benefits [21]. The Theory of Planned Conduct (TPB) holds that information shapes attitudes, which in turn influence behavioral intention and actual behavior. Greater understanding leads to greater utilization of green marketing strategies since they require active engagement in eco-friendly behaviors (such as resource efficiency and sustainable shopping [22]. Thus, the use of green marketing strategies results in a rise in green marketing and its associated aspects. Consequently, the research hypothesized that:

**H1** Nurse managers sharing in the green marketing strategy intervention will display developed levels of green marketing strategy and all related dimensions after the intervention, compared with those in the pre-intervention.

### Green marketing strategy and TQM

According to recent studies, senior management plays a critical role in promoting a sustainable culture in healthcare environments. All staff members must actively participate in the execution of green healthcare initiatives, which are bolstered by ongoing environmental sustainability education and training. According to a South Korean study, healthcare firms can successfully integrate green practices into TQM frameworks, improving operational efficiency and environmental performance [23]. Furthermore, a key paradigm for incorporating sustainability into nursing practice is the idea of Eco-Conscious Nursing. This idea highlights nurses’ need to adopt ecologically conscious practices to improve patient care and environmental health. Nursing may make a substantial contribution to the larger objectives of sustainable healthcare by operationalizing eco-conscious ideas and meeting the industry’s pressing need for eco-friendly methods [24]. In conclusion, the combination of TQM in nursing with green marketing techniques not only encourages sustainable healthcare practices but also improves the standard of patient care. Addressing the issues brought on by climate change and the growing need for ecologically friendly healthcare solutions requires this dual focus.

Total quality management (TQM) in healthcare focuses on productivity, waste reduction, and continuous improvement. It aligns with sustainability goals and allows nurses to implement quality measures for material, waste, and resource efficiency. Nurse managers can implement small-scale improvements through feedback loops. Green marketing can help nurses integrate TQM best practices into daily operations, resulting in improved outcomes and resource efficiency [10&25]. Hence, training nurses on green marketing strategies can improve their TQM towards their labor. therefore, the study hypothesized that:

**H2** Nurse managers who participated in the green marketing strategy intervention will engage in a higher level of TQM after the intervention compared with those who participated before it.

### Green marketing strategy and green performance

Head nurses’ performance is crucial as they interact with healthcare workers daily. Inspiring them can lead to better performance, reduced productivity, and dissatisfaction. A green organizational culture facilitates environmental strategy execution, demonstrating the importance of people’s views. Successful implementation of green marketing techniques enhances performance and competitive advantage [26, 27]. Green marketing tactics in nursing are increasingly recognized for improving environmental performance in healthcare environments. Studies show that incorporating green practices in nursing administration enhances sustainability and organizational effectiveness. Educational interventions can encourage staff to embrace green practices [3].

Integrating green healthcare practices into TQM enhances program efficacy, with top management support and staff involvement crucial. Nursing staff’s ongoing education on sustainability enhances hospital’s environmental performance [2]. Research indicates that environmental attitudes influence consumers’ choices for green healthcare services. Implementing green marketing techniques in nursing homes can improve market position, encourage sustainable practices, and increase customer satisfaction and employee engagement, ultimately promoting environmental sustainability [28]. Hence, green marketing strategy training can improve green performance among nurse managers. therefore, the study hypothesized that:

**H3** Nurse managers participating in the green marketing strategy intervention will exhibit a higher level of green performance post-intervention compared to those in the pre-intervention group.

The Resource-Based View (RBV) theory provides a useful theoretical framework to fill the identified knowledge gap about how green marketing tactics and Total Quality Management (TQM) practices affect green performance among nurse managers. The RBV states that firms can gain a sustained competitive edge by making efficient use of internal resources and capabilities that are valuable, uncommon, unique, and non-replaceable, such as management skills, environmental plans, and quality improvement systems. When used in healthcare settings, TQM best practices (such as staff engagement, continuous improvement, and process optimization) and green marketing strategies (such as promoting eco-friendly practices and services) can be regarded as strategic tools that improve organizational green performance, especially when implemented by proactive nurse managers. By presenting how internal management techniques in nursing might promote sustainability results, RBV’s integration into this setting fills a vacuum in the research [29].

**Fig. 1:**
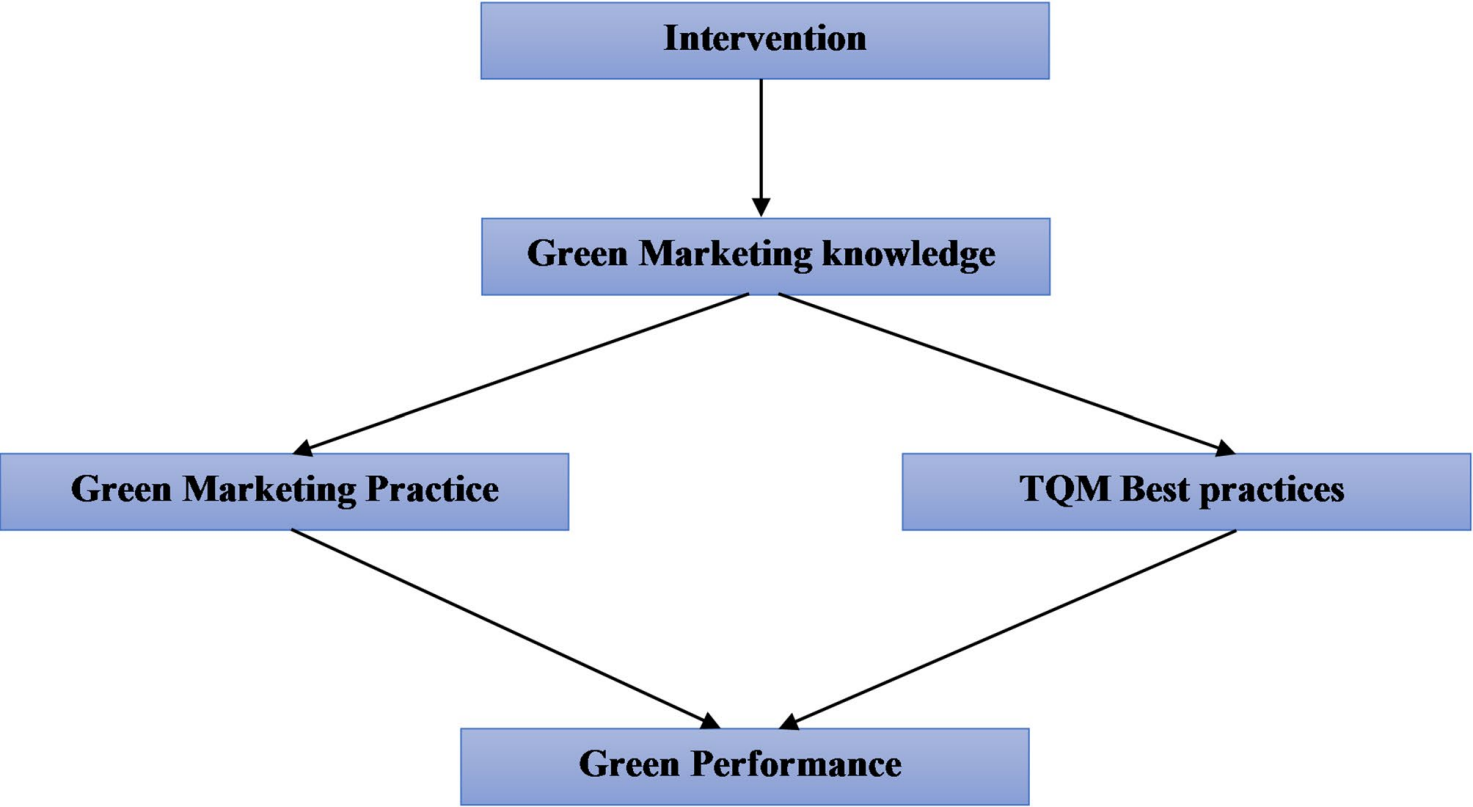
Conceptual model of green marketing strategy intervention

To ensure the utmost clarity and theoretical grounding of our Conceptual Model of Green Marketing Strategy Intervention (Fig. 1), each relationship depicted by an arrow within the model is meticulously supported by the comprehensive methodology outlined in Sect. 3.4, “Intervention.” The development of the green marketing strategy intervention program, based on established green marketing theory, directly informs the hypothesized influences within the model. For instance, the Assessment Phase (3.4.1) provided the empirical basis for identifying initial knowledge and practice levels. At the same time, the subsequent Planning (3.4.2) and Implementation Phases (3.4.3), including the structured training program and the three-week practical application period, directly correspond to the operationalization of the green marketing strategy and its intended effects on various outcomes. The detailed steps for data collection, intervention delivery, and the focus on specific green marketing components (e.g., the 4 Ps) all serve to substantiate the directional arrows, illustrating how the intervention is designed to lead to the desired changes in practice and performance. This rigorous approach ensures that every depicted relationship in Fig. 1 is not merely theoretical but is systematically addressed and explored through our research design and intervention.

## Subjects and methods

### Study design

For one group, a quasi-experimental design consisting of pre- and post-tests was employed. A quasi-experimental research design is an empirical study that falls between correlational studies and real experiments, to estimate the causal effect of an intervention on its target population without randomly assigning participants to the treatment or control group.

### Participants and setting

The study employed a convenience sampling technique due to the specific logistical and practical constraints associated with recruiting nurse managers. Convenience sampling was chosen as it allowed for the inclusion of all available nurse managers who met the inclusion criteria during the data collection period. This method was particularly suitable given the limited number of nurse managers in leadership roles at Shaqra General Hospital and the need to complete the intervention within a defined timeframe. While convenience sampling may limit the generalizability of findings, it was deemed appropriate for this study given its focus on evaluating the specific impact of a green marketing strategy intervention within a localized healthcare setting. Nurse managers with a minimum of two years of experience in leadership roles were targeted to ensure a sufficient level of expertise and familiarity with managerial responsibilities, enhancing the internal validity of the results.

### Sample size

All available nurse managers (*N* = 32) who were on duty at Shaqra General Hospital during the data collection period were included in the study. To confirm the adequacy of the sample size, a post-hoc power analysis was conducted using G*Power version 3.1.9.7. The analysis was performed with the following parameters: an effect size dz = 0.8 (large), an alpha level of 0.05, a total sample size of 32, and a two-tailed test. The resulting statistical power (1 − β) was 0.94, exceeding the standard threshold of 0.80. These results indicate that the sample size was sufficient to detect significant differences for the main study outcomes with a large effect size. While the sample size may limit generalizability, it was appropriate for the study’s quasi-experimental design and focus on a specific healthcare setting.

Using a convenience sample technique, all available nurse managers who were on duty at their place of employment at the time of data collection were included in the research N= (32) nurse managers.

### Intervention

The concepts of the green marketing theory served as the foundation for the development and operationalization of the green marketing strategy intervention program. The green marketing theory influenced the green marketing strategy intervention program, aiming to provide participants with a long-term competitive edge. Marketing supports business expansion by attracting and retaining clients, focusing on meeting client needs. It also guides immediate marketing initiatives and assists nurse managers in persuading patients.

The implementation of a Green Marketing Strategies training program was based on the model for a training program. Three phases were defined: the preplanning and assessment phase, the action training phase, and the follow-up phase. The data collection occurred from February 2023 to the beginning of May 2023.

#### Assessment phase

1. In the preplanning and assessment phase, authoritative personnel granted permission to conduct the study before starting the study. The participants provided verbal consent.
2. To gain a better understanding of the work environment, interviews with potential participants and nurse managers at different levels were conducted prior to the intervention program to understand the work context, which included the following: what they believed to be good performance, obstacles to their ability to perform effectively, resources that help them provide high-quality care, difficult tasks, and reasons for not taking on such challenges were among the topics covered in the interview. A customized intervention and examples that were especially suited to the circumstance were created using this information.
3. It was requested that recommendations regarding the structure and content of the intervention materials be made by a five-person expert panel consisting of two post-doctoral staff nurses, one nursing manager, and two professors of nursing administration. The intervention materials were revised and resend to the committee for approval in accordance with their suggestions. To ensure that the intervention materials were high-quality and clear, pilot testing was conducted on them.
4. As an initial assessment of the nurse managers, knowledge about Green Marketing Strategies was assessed using the Self-administered Green Marketing Knowledge Questionnaire. Concurrently, nurse managers complete the green marketing practice, TQM questionnaires, and nurse managers performance assessment scale. This was completed from February 1, 2023, to February 15, 2023. Each nurse manager spent about 20–25 min answering the questionnaires.

#### Planning phase

1. The results obtained from the initial assessment of nurse managers’ knowledge and green marketing practice were analyzed. Then, the educational needs were delineated. Accordingly, a training program was designed as well as a training program schedule. The training program was implemented for nurse managers. The green marketing training program took place from February 2023 to March 1, 2023.

#### Implementation phase

Three groups of nurse managers were selected to receive the training program based on their departments. First, all supervisors in the safety, infection control, and quality departments received training. One head nurse from each department was part of the second training group. One head nurse from various hospital departments made up the third training group. The manager of each group of nurses received one week of training during the three weeks when the training programs were in place. Each group received two-hour training sessions four times a week.

Thereafter, the intervention was conducted, consisting of the intervention, or interviewing which was followed by a recovery session, three weeks of implementing the green marketing plan, and a two-day training. The two-day course was divided into four sixty- to ninety-minute sessions, with a thirty-minute intermission. In session one, the researcher provided an outline of the theoretical background. In addition to a thorough explanation of the green marketing theory and methodology. In session two, the strategies for green marketing were discussed as well as the barriers to sustaining green marketing. In session three, the marketing mix’s goal 4 Ps (product, pricing, place, and promotion green marketing) in green marketing were discussed. In session four, a personal green marketing plan and the role of nurse managers in green marketing were discussed. Each session began with the establishment of objectives and an explanation of the new topic, followed by a discussion of the questions posed by the nurse managers. The workshop’s themes are listed in full in Table 1.

After the workshop, each participant received a booklet including the instructional materials. These resources were developed taking into account the needs of the nurse managers as determined by interviews and a thorough review of the literature [30].

The program was implemented in the conference rooms of the hospital for nurse managers. The following teaching methods were employed: brainstorming, discussion, small-group work, and lectures.

After the session, the participants had to put green marketing strategies into practice for three weeks while following the researchers’ developed plan and a list of green marketing strategies. After that, in weeks 1, 2, and 3, participants were told to concentrate on tasks related to increasing resources and overcoming difficulties. During the three-week intervention phase, the participants were encouraged to join a WhatsApp group that was set up to provide support, mentorship, experience sharing, and reminders twice a week to engage in the prescribed behaviors.

Following the completion of the three-week intervention program, a reunion meeting was scheduled. Participants in this session were asked to evaluate their approaches to product marketing, taking into account their prior successes, obstacles they had encountered, and solutions they had come up with. After the meeting, the researcher expressed gratitude to each member of the group.

In the end, the best way to acquire a sustained competitive advantage is through a solid marketing strategy. It comprises identifying target markets, creating compelling value propositions, leveraging the marketing mix deftly, and synchronizing campaigns with organizational objectives. Organizations that use strategic green marketing are better equipped to attract clients, boost revenue, build loyal fans, and keep a competitive edge in a market that is always changing.

#### Evaluation phase

A green marketing knowledge questionnaire, the green marketing practice scale, the nurse managers TQM practice questionnaire, and the Nurse Manager Performance Assessment Scale (NMPAS) were distributed again after three months of the program to compare with the pre-test and to evaluate the program effect size on TQM practice and nurse managers performance. The follow-up period was filled in March 2023.

**Table 1:** The workshop’s themes

Sessions	Goals	Activities
**First session** **(Theoretical background)**	• Establish the group • Introduce the program • Introduce the concept of green marketing strategy. • Distinguish between marketing and green marketing strategy • Explore the importance of green marketing strategy • Discuss Types of Marketing Strategies (Traditional Marketing Strategies and Digital Marketing Strategies)	(i) Warm-up game and acquaintances (ii) Establishment of a group contract (iii) Clarification of the program’s aim, activities, and timeline (iv) Explanation of the concepts of green marketing by the researcher (v) Explore the importance of green marketing strategy. (vi) Describe the Types of Marketing Strategies (Traditional Marketing Strategies and Digital Marketing Strategies).
**Second session** (8 strategies for green marketing, barriers to sustaining green marketing)	1) Focus on the 8 strategies for green marketing 2) Examples of Green Marketing Strategies (**Patagonia**, **Tesla**, and **Unilever**).	(i) Lecture on 8 strategies for green marketing (ii) Provision of examples of Green Marketing Strategies (**Patagonia**, **Tesla**, and **Unilever**). (iii) Sharing of personal Green Marketing Strategies experiences in terms of producing sustainable products, using sustainable materials to make products, and seeking challenges with each other by the participants (iv) Brainstorming about barriers to sustaining green marketing (v) Review of the day (vi) Provision of homework that aims to identify products and challenges other than those mentioned in the session
**Third session** Discussion of the marketing mix’s goal 4 Ps	1) Complete the Influence of Green Marketing Strategies 2) Challenges and Considerations 3) Identify the Marketing Mix: The 4 Ps (Product, price, Place (Distribution), and promotion). 4) Pro Tips for Green Marketing	(i) Sharing homework from the previous session (ii) Brain storming about product and promotion they have in their workplace (iii) Group discussion about tasks which represent job challenges (iv) Participants summarize individually their own strengths, motive, weakness point and obstacles, they experience in their work and matched (v) Group discussion about Marketing Mix: The 4 Ps (Product, price, Place (Distribution), and promotion). (vi) exploration the Influence of Green Marketing Strategies Create Pro Tips for Green Marketing
**Fourth session** **(Personal green marketing plan and** Role of nurse manager in green marketing)	1) Create the personal green marketing plan 2) Clarify role of nurse manager in green marketing) 3) Clarify the next intervention	(i) Participants individually formulating their own green marketing goals and activities they could practice to maximize challenges and optimize green marketing strategy to carry out in the upcoming three weeks (ii) Discuss role of nurse manager in green marketing (iii) Discussion about the upcoming application (iv) Concluding all sessions

### Measures

The study measures included scales that measure the practice of green marketing in recently and developed countries questionnaire, self-administered Knowledge about Green Marketing, demographic information form, Total Quality Management Practice Survey, Nurse Manager Performance Assessment Scale (NMPAS), and the researchers used the committee approach to translation the scales, which were first created in English, into Arabic [31]. Four members of the committee independently and concurrently translated the scales. Four academic staff members from the university’s nursing department evaluated the translated tools. The panel also looked at the measurement instruments for face and content validity as well as back translation from Arabic to English. required adjustments in compliance with the panel’s suggestions. Using reliability analysis, the instrument’s internal consistency was evaluated.

#### Primary outcome

**(1) The practice of green marketing in newly and developing countries questionnaire**: [32] Soussa (2001) designed this self-administered questionnaire. Given the varied cultural backgrounds of the individuals and the environment, the researcher modified the instrument in response to expert editing and modification, jury criticism, and clarifications from the pilot study. This tool aims to examine the opinions of all nurse managers about green marketing practices in a Saudi Arabian governmental hospital. This contained 85 items divided into 4 dimensions namely Marketing Orientation (20 items), Marketing Activity (30 items), Marketing Functions (26 items), and Marketing Development (9 items). Each statement answer was measured on a five-point Likert scale that ranged from 1 = strongly disagree to 5 = strongly agree. In its original version, the Cronbach’s alpha for scale dimensions is 0.69–0.76. In our study, its reliability across the two-time points was acceptable (**0.74**).

**Practice of green marketing in new and developing countries questionnaire scoring system**: Three categories were identified by [33] cut of point scoring system: low level of nurse managers opinions about green marketing practice (less than 50%), moderate level of opinions about green marketing practice (between 50 and 75%), and high level of opinions about green marketing practice (more than 75%).

#### Secondary outcomes

***(1)*** The Green Marketing Knowledge Questionnaire (GMKQ) was formed by the researcher using a review of relevant literature as a basis [34–36]. The nurse managers’ understanding of green marketing was assessed using this tool both before and after an educational intervention. Consists of 15 questions divided between 6 multiple-choice questions and 9 true-false questions. There were a set of questions for each program session. Examples of such questions include those about marketing definitions, marketing importance, marketing process, marketing mix model, types of markets, marketing components, marketing practitioners, and other aspects of the nurse managers’ involvement in healthcare marketing.

##### Green marketing knowledge questionnaire scoring system

15 questions that had higher scores indicate more knowledge; the score ranges from 0 to 15. Two criteria make up the scoring system according to [37] (1) Poor knowledge (less than 60%). (2) Sufficient understanding (60+%).

**(2) Total quality management practice survey** The purpose of a self-administered questionnaire sheet created by [38] Lee (2010) is to investigate nurse managers’ perceptions of TQM practice. 42 elements total, separated into six dimensions: information analysis (7 items), process management (7 items), customer focus (7 items), human resource emphasis (7 items), leadership (7 items), and strategic planning (7 items). Every response to a sentence will be evaluated using a five-point Likert scale, where 1 represents strongly disagree and 5 represents strongly agree.

**Total quality management practice survey scoring system**: Three categories were identified by [33] the cut-off value scoring system: low level of nurse managers opinions about overall quality management practice (less than 50%), moderate level of nurse manager opinions about overall quality management practice (between 50 and 75%), and high level of nurse manager opinions about overall quality management practice (more than 75%). the Cronbach’s alpha in its original version was 0.79. In this study, the reliability across the two- time points were acceptable (**0.73**).

(3) **Nurse manager green performance assessment scale (NMGPAS)**: this tool was developed by [39] KOÇ ASLAN, et al., (2023). It will be applied to nurse managers’ green performance evaluations. The instrument consists of nine items and Seventy-one criteria. These items are: “Leadership “Track and control (Providing the necessary guidance and support with which to achieve the goals (7 criteria), “Self-management (Effective management of personal impact and image by demonstrating and developing the necessary knowledge/skills (14 criteria), “Team management (Effective management of relationships with team members, 11 criteria), “Determining the method (Planning the tasks that need to be performed in order to achieve the department’s goals, taking into account the priorities of the institution, (13 items), “Improving efficiency and productivity (Regular evaluation of the effectiveness and efficiency of practices and creating development opportunities (3 criteria), “Preparing required resources (Creating the necessary arrangement and preparing the necessary resources with which to execute the plan, (7 criteria), “Setting a goal (Determination of department objectives that will support the achievement of the institution’s vision, 5 criteria), planning and evaluation (4 items), and “Management of external partners’ relations (Working with partners outside of the team, including the first manager, managers of cooperating teams, key customers to whom services are offered, and key suppliers from which services are received; establishment and effective management of relationships, 7 items). These are the nine main dimensions under which it is organized. Each activity on the observation checklist is given a yes, no, or not applicable score. “Yes” received a point, “no” received zero, and “not applicable” was not included in the computation.

The following scoring guidelines were applied by [40] < 50% bad performance, 50%–< 65% moderate, 65%–< 75% good, and 75%–< 85% extremely good. 85–100% outstanding output. Cronbach’s alpha in its original version was 0.85. In this study, the reliability across the two-time points was acceptable (0.736).

#### Demographic information form

This form consists of questions related to seven personal data items, along with gender, age, marital status, education, employee number, experience year, and occupational levels.

### Validity

Five professionals with backgrounds in academia and clinical settings validated the study’s intervention program and data collection tools. In all tools of data collection, the Factor loadings were acceptable assessed, and confirmed to be above 0.40, supporting the questionnaire’s dimensional structure.

### Pilot study

A pilot study was carried out 10% to evaluate the quality and clarity of the intervention materials, the length of time needed for data collection, and the feasibility, validity, and reliability of the study measures. Three nurse managers who fulfilled the inclusion and exclusion criteria but were not part of the study population took part in the pilot trial. The study’s findings demonstrated that the intervention materials and study measures were intelligible and that no changes were necessary.

### Data analysis

The statistical analysis was conducted using SPSS version 28. Non-parametric tests were employed due to the small sample size and the non-normal distribution of the studied variables. The Mann-Whitney U test was used to compare differences between two independent groups, such as gender, marital status, education level, and occupational level, with respect to knowledge, the practice of green marketing strategies, total quality management practices, and nurses’ performance. The Kruskal-Wallis H test was applied to compare differences among more than two independent groups, such as age and years of experience, for the same variables.

Spearman’s rank correlation coefficient was used to examine the relationships between continuous variables, including knowledge, the practice of green marketing strategies, total quality management practices, and nurses’ performance before the intervention. To assess the differences in pre- and post-intervention scores, the Wilcoxon Signed Ranks test was applied for all studied variables, including knowledge, the practice of green marketing strategies, total quality management practices, and nurses’ performance across various domains. A significance level of *p* < .05 was considered statistically significant for all tests.

### Ethics consideration

The study procedure (ID/Zu.Nur.REC#:0 Date 21/02/2024) was approved by the Research Ethics Committee of the Faculty of Nursing at Zagazig, Egypt. Also, ethical approval was obtained from the standing committe of scientific research ethics at Shaqra University, Saudia Arabia (ERC-SU-S-202500124). All necessary information about the study was introduced in the first section of the sheet. The questionnaire included a statement related to the aim and nature of the study. All participants who chose the word agree to give their informed consent before beginning their response to the sheet. The respondents were guaranteed the privacy and confidentiality of their answers, the voluntary nature of their involvement, and the fact that their absence would not hurt their grades or result in any negative outcomes. Participants have given their informed consent under the criteria outlined in the Helsinki Declaration. It was determined that participants had the right to withdraw from the study at any time.

## Results

Table 2 Contains the demographic characteristics of the nurse managers and shows that most of the participants were female (84.4%). Also, most of them were < 30 years (75%) while the few studied nurse managers from 30 - >45 years (8%). Most of the participants (53.1%) were married, and most of them had baccalaureate degrees of education (78.1%). Also, most of the participants had < 30 years in employee No and most of them were from 1 to 5 years of experience (59%), while a few of them had > 10 years of experience (9.4%) respectively. regarding occupational level, most of the participants were head nurses (81.3%).

**Table 2:** Demographic information of studied groups of nurse managers (*n* = 32)

Variables	Frequency		Difference			
	No	%	Knowledge	Practice	QMP	OC
Gender						
Female	27	84.4%	Z = 1.738	Z = 0.467	Z = 0.676	Z = 0.366
Male	5	15.6%	P=,082	*P* = .640	*P* = .499	*P* = .366
Age						
< 30	24	75%	Z = 0.155	Z = 1.045	Z = 0.785	Z = 1.759
30->45	8	25%	*P* = .877	*P* = .296	*P* = .433	*P* = .073
Marital s						
Single	15	46.9%	Z = 0.690	Z = 0.888	Z = 1.835	Z = 0.656
Married	17	53.1%	*P* = .490	*P* = .375	*P* = .067	*P* = .512
Education						
Bachelor’s degree	25	78.1%	Z = 0.578	Z = 0.091	Z = 0.685	Z = 0.816
Master degree	7	21.9%	*P* = .563	*P* = .927	*P* = .493	*P* = .414
Employee No						
< 30	24	75%	Z = 0.663	Z = 0.392	Z = 0.283	Z = 1.842
30->45	8	25%	*P* = .508	*P* = .695	*P* = .777	*P* = .065
Experience years						
1–5 years	19	59.4%	H = 1.046 *P* = .593	H = 2.999 *P* = .223	H = 0.805 *P* = .669	H = 2.785 *P* = .249
6–10 years	10	31.3%				
> 10 years	3	9.4%				
Occupational level						
Head nurse	26	81.3%	Z = 0.490	Z = 1.666	Z = 1.669	Z = 1.625
Supervisor	6	18.8%	*P* = .624	*P* = .097	*P* = .095	*P* = .104

Z = Mann-Whitney U, H = Kruskal-Wallis H

Table 3 descriptive statistics of studied nurse managers and this table showed that It highlights significant improvements across all measured dimensions. For instance, the mean Total Knowledge increased markedly from 6.37 to 14.56, while the Practice Total rose from 253.81 to 344.59, indicating substantial knowledge and practical application growth. Similarly, the QMP Total and performance Total showed considerable improvements, with mean values increasing from 123.25 to 186.34 and 67.62 to 138.06, respectively. Additionally, these trends strongly indicate the effectiveness of the intervention in enhancing performance and reducing variability in the results.

**Table 3:** Descriptive statistics of studied groups of nurse managers (*n* = 32)

Descriptive Statistics					
Studied variable	*N*	Min	Max	Mean	S.D
Total Knowledge	32	1	9	6.37	1.85
Total green marketing Practice	32	85	365	253.81	64.25
Total Quality Management practice	32	42	210	123.25	40.14
Total nurse manager green performance	32	36	73	67.62	8.05
Total. Knowledge. Post	32	12	15	14.56	0.80
Total green marketing Practice. Post	32	260	415	344.59	34.95
Total Quality Management Practice. Post	32	145	206	186.34	12.24
Total nurse manager green performance. Post	32	132	142	138.06	3.40

Table 4 demonstrated significant differences between the studied domains and total scores of the variables pre- and post-interventions. The median score for total green marketing knowledge increased significantly from 6 (range 1–9) to 15 (range 12–15) with a Z value of 4.950 and a p-value of < 0.001. All aspects of the practice of green marketing strategies also showed significant improvements, with substantial increases in median scores across marketing orientation, marketing activity, marketing functions, and marketing development, all with p-values < 0.001. The total practice of green marketing strategies showed a significant increase from 251 (range 85–365) to 352 (range 260–415) (Z = 4.735, *p* < .001).

**Table 4:** Score differences between studied domains and total scores of studied variables related to knowledge of green marketing and green marketing strategies

Variables	Median (range)		Test of Significance	
	Pre	post	Z	*P*
Total Knowledge Marketing Knowledge	6 (1–9)	15 (12–15)	4.950	< 0.001
The Practice of Green Marketing Strategies				
Marketing Orientation	59 (20–78)	84 (60–99)	4.938	< 0.001
Marketing Activity	88 (30–133)	129.5 (85–148)	4.694	< 0.001
Marketing Functions	78.5 (26–128)	105.5 (69–130)	2.890	0.004
Marketing Development	25.5 (9–45)	35.5 (9–45)	2.782	0.005
Total Practice Green Marketing Strategies	251 (85–365)	352 (260–415)	4.735	< 0.001

Z = Wilcoxon Signed Ranks Test, *Significant at a level less than 5% (*p* < .05), **Significant at a level less than 1% (*p* < .01)

Table 5 Similarly, Score differences between studied domains and total scores of studied variables related to TQM practice. The total quality management practices displayed notable improvements in all subdomains, including leadership and management, strategic planning, customer focus, human resource focus, process management, and information analysis, with all Z values indicating significance at *p* < .001. The overall total quality management practice score significantly increased from 125 (range 42–210) to 188.5 (range 145–206) (Z = 4.834, *p* < .001).

**Table 5:** Score differences between studied domains and total scores of studied variables related to TQM practice

Variables	Median (range)		Test of Significance	
	Pre	post	Z	*P*
Total Quality Management Practice				
Leadership & Management	21 (7–35)	31.5 (22–35)	4.668	< 0.001
Strategic planning	21 (7–35)	33 (28–35)	4,809	< 0.001
Customer focus	21 (7–35)	34 (15–35)	4.265	< 0.001
Human Resource Focus	20 (7–35)	31 (20–35)	4.682	< 0.001
Process management	20.5 (7–35)	33.5 (21–35)	4.845	< 0.001
Information Analysis	21 (7–35)	28 (17–34)	3.942	< 0.001
Total Quality Management Practice	125 (42–210)	188.5 (145–206)	4.834	< 0.001

Z = Wilcoxon Signed Ranks Test, *Significant at a level less than 5% (*p* < .05), **Significant at a level less than 1% (*p* < .01)

Table 6 Finally, Score differences between the studied domains and total scores of the studied variables related to nurse managers’ green performance. nurses’ manager performance scores also improved across all subdomains, such as leadership (Track and control), “Self-management, “Team management, “Determining the method, “Improving efficiency and productivity, “Preparing required resources, “Setting a goal, planning and evaluation, and “Management of external partners’ relations, with Z values ranging from 5.018 to 5.386 and p-values < .001. The total score for nurses’ performance increased significantly from 71 (range 36–73) to 139 (range 132–142) (Z = 4.940, p < .001). Overall, the intervention led to statistically significant improvements across all variables and domains.

**Table 6:** Score differences between studied domains and total scores of studied variables related to nurse managers’ green performance

Variables	Median (range)		Test of Significance	
	Pre	post	Z	*P*
Leadership (tracking ad control)	7 (2–9)	14 (11–14)	5.047	< 0.001
Self-management	14 (5–14)	28 (25–28)	5.065	< 0.001
Team management	11 (2–12)	22 (18–22)	5.171	< 0.001
Determining the method	13 (7–13)	26 (23–26)	5.018	< 0.001
Improving efficiency and productivity	3 (0–4)	6 (5–6)	5.386	< 0.001
Preparing required resources	7 (4–7)	14 (12–14)	5.333	< 0.001
Setting goal	5 (3–6)	10 (6–10)	5.074	< 0.001
Planning and evaluation	4 (1–5)	8 (5–8)	5.122	< 0.001
Management of external partners’ relations	7 (3–11)	14 (13–14)	5.286	< 0.001
Total of Nurses manager green Performance	71 (36–73)	139 (132–142)	4.940	< 0.001

Z = Wilcoxon Signed Ranks Test, *Significant at level less than 5% (*p* < .05), **Significant at level less than 1% (*p* < .01)

**Fig. 2:**
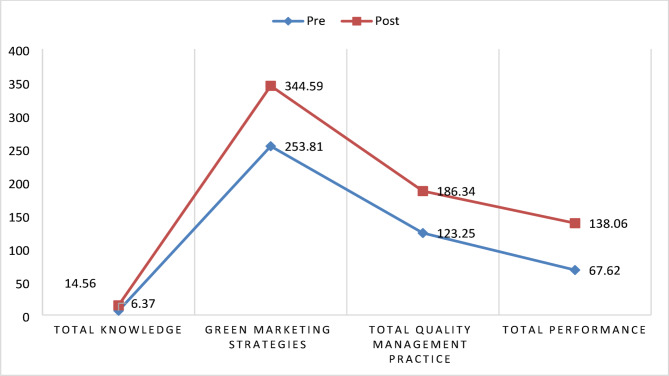
Total knowledge of green marketing, green marketing practice, TQM, and total performance of nurses’ managers pre and post-program intervention

Figure 2. This figure illustrates the improvement in total knowledge of green marketing, green marketing practices, TQM, and total green performance among nurse managers following the post-program intervention compared to the pre-program intervention.

### Hypothesis testing outcomes

The study’s hypotheses were tested by comparing the pre- and post-intervention scores of the nurse managers. The results from the Wilcoxon signed-rank tests provide clear evidence for each hypothesis.

**Hypothesis 1 (H1)** This hypothesis predicted that nurse managers participating in the green marketing strategy intervention would display developed levels of green marketing strategy and all related dimensions after the intervention. The results strongly support this hypothesis. There was a statistically significant increase in the median score for total green marketing knowledge from 6 to 15 (Z = 4.950, *p* < .001). Similarly, the total practice of green marketing strategies score rose significantly from a median of 251 to 352 (Z = 4.735, *p* < .001). These findings confirm that the intervention was effective in enhancing both knowledge and practice, thereby supporting H1.

**Hypothesis 2 (H2)** This hypothesis posited that nurse managers who participated in the intervention would engage in a higher level of Total Quality Management (TQM). The data corroborate this hypothesis. A significant improvement was observed in the overall total quality management practice score, which increased from a median of 125 pre-intervention to 188.5 post-intervention (Z = 4.834, *p* < .001). This demonstrates that the green marketing intervention had a positive spillover effect on TQM practices, supporting H2.

**Hypothesis 3 (H3)** This hypothesis anticipated that nurse managers would show a developed level of green performance after the intervention. The results provide robust support for this hypothesis. The total score for nurse managers’ green performance increased significantly from a median of 71 to 139 (Z = 4.940, *p* < .001). This outcome indicates that the program successfully translated into improved green performance in their roles, thus supporting H3.

Overall, the statistical analysis confirms that all three hypotheses were supported by the data collected, indicating that the green marketing strategy intervention led to significant improvements in nurse managers’ knowledge and practice of green marketing, TQM engagement, and green performance.

## Discussion

As a supporting management tool for enhancing organizational performance, every organization should consider total quality management and green marketing strategies. Perhaps the most effective management strategy used by businesses to improve the quality of their goods and services and raise standard metrics of overall business performance (such as increased revenue, market share, and lower costs) is total quality management. Therefore, this study aimed to evaluate green marketing strategies and best practices of TQM on green performance among nurse managers.

The result of our study showed that nurse managers who underwent the intervention program reported significantly higher levels of green marketing knowledge after the intervention, with a large effect size. this finding is valuable because it indicates that green marketing strategy is a trainable behavior. This enhancement might be the result of lectures that are delivered in a clear, succinct, and easy-to-understand manner, as well as the availability of pertinent media that provides additional examples to help nurse managers understand the material and inspire them to participate in the program. Additionally, the pleasant interactions throughout program sessions demonstrated that the nurse managers were interested in the content of the program. These results are supported by the findings of [41] that highlight the significance of sustainability education programs for nurses. These programs have been shown to improve nurses’ knowledge and competence, change their attitudes, develop their problem-solving and interpersonal skills, and inspire them to work harder.

Also, these findings are consistent with [42] past research that demonstrates how incorporating green marketing strategies into an organization may boost organization sustainability and competitiveness while also satisfying the growing expectations of consumers for eco-friendly products. These strategies are determined to be essential for businesses to successfully handle the environmental and market issues of today. Additionally [43], concur with this outcome and state that businesses who are up against fierce competition should benefit from this by putting green marketing strategies into practice. Managers must understand the significance of an ecologically friendly marketing strategy in order to boost consumer happiness, according to [15] they also noted that the absence of a green marketing strategy has a major negative impact on nurse managers satisfaction.

The results of the study show that the total quality management practices displayed notable improvements in all subdomains, including leadership and management, strategic planning, customer focus, human resource focus, process management, and information analysis. The overall total quality management practice score significantly increased after the implementation of the program. The increased of TQM practice caused by the implementation of GMS program. The outcome is in line with [23] The study’s findings demonstrated that, Top management plays a crucial role in implementing green healthcare initiatives, encouraging active participation, providing ongoing education, and closely monitoring organizational progress. Additionally, this outcome supports the findings of [33] who found that nurse managers’ marketing practices and understanding improved statistically considerably after the program intervention. Additionally, the nurse manager’s overall total quality management practice demonstrated a statistically significant improvement after the program was implemented, they concluded that the implementation of a marketing training program increased nurse managers’ evaluations of overall quality management techniques and their degree of marketing understanding.

The result of the present study showed that nurse managers green performance scores also improved across all subdomains, such as leadership, Self-management, “Team management, “Determining the method, “Improving efficiency and productivity, “Preparing required resources, “Setting a goal, planning and evaluation, and “Management of external partners ‘relations after the program implantation, resulting in their becoming more skilled and effective in their workplace. This result agrees with [43] according to research, implementing a green marketing strategy enhances an organization’s performance by providing product distinctiveness, enhancing its reputation and image, and boosting sales of environmentally friendly goods. Businesses can perform better when the government implements green legislation. As a result, the organization’s performance improves by its green marketing plan. Furthermore [44], research revealed that the green marketing approach positively affects green performance through competitive advantage. Lastly, through competitive advantage, competitive intensity mitigates the indirect impact of green marketing strategy on (GP). Additionally, a green marketing strategy directly improves (GP), indicating a partial mediation.

According to research by [45], green marketing has a major positive impact on enhancing green environmental performance, as shown by decreased carbon footprint, effective waste management, and sustainable sourcing. Additionally, these strategies improve consumer perception, brand loyalty, and sales growth, all of which have a beneficial impact on market performance [46]. according to the report, a green marketing approach improves an organization’s performance and organization image. The essay suggests using green marketing strategies by businesses to keep a competitive advantage.

The study findings showed that there was a statistically significant improvement in total nurse managers’ green marketing knowledge, total green marketing practice, TQM, and total nurse managers’ green performance in all sessions when comparing the pre-program stage with post- program. This improvement was due to the effect of the intervention program; attendance of the program affects positively on nurse managers marketing knowledge. One explanation for this is that nurses with advanced degrees and years of experience would have honed the practice knowledge, skills, and abilities necessary for increased dedication and performance. This is in the same line with [47] who added that providing knowledge and practical mechanisms to boost environmental protection and promote sustainable development is critical. These findings were supported by the findings of [48] They concluded that TQM, when combined with effective knowledge application, facilitates sustainable practices, promotes continuous improvement, and aids in efficient management and utilization of knowledge resources, leading to enhanced performance. Also, these findings are congruent with [25] its findings that TQM intervention may help promote continuous improvements in advancing cost-effective quality care, this study enriched the existing body of knowledge in the TQM, nursing, and health management research field. In addition [2], concluded that more environmentally conscious nurses were more likely to practice sustainable practices like cutting back on waste, conserving energy, and making eco-friendly purchases (*p* < .05). Moreover, these findings are in line with [49] They claimed that TQM procedures significantly contribute to achieving (GP) objectives in the medical field, demonstrating that even in poor nations, organizations can enhance their green marketing through proper application. also [50], disputes the findings of the study which indicated that green performance (GP) is highly impacted by total quality management (TQM).

This result disagrees with [51], which underlined that although knowledge is crucial, other elements like organizational culture and leadership may also influence green marketing behavior. Also [52], This disagrees with the weak, non-significant correlation you discovered between TQM and knowledge (*r* = .211, *p* = .246), suggesting that although knowledge plays a role, other elements like leadership and resources are also quite important [53]. Choi and Park investigated the connection between performance and knowledge in healthcare environments. They discovered a weak but positive association between knowledge and performance, which is contrary to your findings (*r* = − .008, *p* = .964). They attribute this to the fact that information alone cannot guarantee [9].

Last but not least, the current study showed that, following the implementation of a green marketing program for nurse managers, the program enhances the knowledge of the staff related to GMS, skills, and overall quality management of the nurses’ managers as well as their green performance in a Saudi Arabian governmental hospital. Therefore, nurse leaders should create a green marketing plan for the healthcare services their department offers, as well as a strategy for how to develop and enhance it to maintain high standards of care and win over patients.

## Conclusion and recommendations

A quasi-experimental design showed that in the context of the green marketing strategy, the green marketing intervention, which involves workshops and green marketing activities, there was a significant positive relationship between the practice of green marketing strategies and total quality management practices which improve the green performance of nurse managers.

Enhancing green performance among nurse managers necessitates the integration of green marketing strategies and Total Quality Management (TQM) principles. This theoretical intersection underscores the importance of embedding sustainability within healthcare administration, thereby aligning quality improvement initiatives with environmental objectives. By leveraging green marketing strategies, nurse managers can effectively communicate the significance of sustainability to both employees and patients, fostering a culture of environmental responsibility. This approach not only strengthens the institution’s reputation but also addresses the increasing demand for eco-friendly healthcare practices.

From a practical perspective, the application of TQM principles such as staff engagement, customer-centricity, and continuous improvement plays a crucial role in advancing green performance. Implementing these principles enhances resource efficiency, reduces waste, and optimizes operational processes, contributing to a more sustainable healthcare environment. The tangible outcomes of these initiatives, including reduced energy consumption and improved waste management, are vital in achieving sustainability targets.

The primary contribution of this research lies in its structured approach to examining the synergy between TQM and green marketing in promoting sustainable nurse management practices. By providing a comprehensive theoretical framework, this study facilitates the development of best practices that not only enhance environmental performance but also elevate healthcare quality. Given their dual focus on sustainability and quality, nurse managers emerge as key drivers in the transition toward greener healthcare systems, ultimately benefiting both the organization and the broader community.

This study highlights the significance of authentic green marketing initiatives and presents several key recommendations: Hospital administrators should develop targeted marketing strategies tailored to different hospital sectors. Hospital managers must remain vigilant about market dynamics and be prepared to adapt proactively. Nurse managers should recognize and address the professional development, autonomy, and achievement needs of staff nurses by facilitating access to workshops and continuous education programs. The marketing committee should include a healthcare marketing expert to enhance its effectiveness, with consideration given to revitalizing its role based on marketing audits. Strategies such as conferences and workshops should be employed to raise awareness among nursing staff regarding the implications of green marketing strategies. Healthcare services should be diversified to meet the specific needs of various market segments.

### Implications for nursing management

This study’s conclusions have several real-world applications. First, creating best practices for green healthcare initiatives could be facilitated by the study’s findings. Second, the results of this study offer comprehension and information regarding the prerequisites (such as involvement activities, training and education, and monitoring) needed to achieve green healthcare. Finally, campaigns that assist healthcare organizations’ adoption of green healthcare practices might make use of the green performance that this study established. Campaigns like this can encourage collaboration between communities and stakeholders to advance green healthcare initiatives.

### Research limitations

The study included a focused sample of 32 nurses and managers from Shaqra Government Hospital, providing in-depth insights relevant to similar healthcare settings. To enhance the generalizability of these findings, future research could involve larger samples from diverse regions to build upon and validate these results.

### Directions for future research

Future studies should include a larger and more diverse sample of nurses and managers across multiple hospitals and regions to improve generalizability and account for variations in healthcare settings. Utilizing both qualitative and quantitative methodologies can provide a more comprehensive understanding of the factors influencing nursing management effectiveness. Investigating the role of digital health solutions, such as electronic health records and AI-driven scheduling, in improving managerial efficiency and reducing workload burdens.

## Data Availability

The data that support the findings of this study are available from the corresponding author upon reasonable request.
